# Mitochondrial and Nuclear Ribosomal DNA Evidence Supports the Existence of a New *Trichuris* Species in the Endangered François’ Leaf-Monkey

**DOI:** 10.1371/journal.pone.0066249

**Published:** 2013-06-20

**Authors:** Guo-Hua Liu, Robin B. Gasser, Peter Nejsum, Yan Wang, Qiang Chen, Hui-Qun Song, Xing-Quan Zhu

**Affiliations:** 1 State Key Laboratory of Veterinary Etiological Biology, Key Laboratory of Veterinary Parasitology of Gansu Province, Lanzhou Veterinary Research Institute, Chinese Academy of Agricultural Sciences, Lanzhou, Gansu Province, People’s Republic of China; 2 Faculty of Veterinary Science, The University of Melbourne, Melbourne, Victoria, Australia; 3 Departments of Veterinary Disease Biology and Basic Animal and Veterinary Science, University of Copenhagen, Copenhagen, Denmark; 4 College of Veterinary Medicine, Hunan Agricultural University, Changsha, Hunan Province, People’s Republic of China; 5 Guangzhou ZhongDa Medical Equipment Co., Ltd., Guangzhou, Guangdong Province, People’s Republic of China; BiK-F Biodiversity and Climate Research Center, Germany

## Abstract

The whipworm of humans, *Trichuris trichiura*, is responsible for a neglected tropical disease (NTD) of major importance in tropical and subtropical countries of the world. Whipworms also infect animal hosts, including pigs, dogs and non-human primates, cause clinical disease (trichuriasis) similar to that of humans. Although *Trichuris* species are usually considered to be host specific, it is not clear whether non-human primates are infected with *T. trichiura* or other species. In the present study, we sequenced the complete mitochondrial (mt) genome as well as the first and second internal transcribed spacers (ITS-1 and ITS-2) of *Trichuris* from the François’ leaf-monkey (langur), and compared them with homologous sequences from human- and pig-derived *Trichuris*. In addition, sequence comparison of a conserved mt ribosomal gene among multiple individual whipworms revealed substantial nucleotide differences among these three host species but limited sequence variation within each of them. The molecular data indicate that the monkey-derived whipworm is a separate species from that of humans. Future work should focus on detailed population genetic and morphological studies (by electron microscopy) of whipworms from various non-humans primates and humans.

## Introduction

Neglected tropical diseases (NTD_S_) have a devastating effect on animal and human health and food production globally. For instance, it is estimated that more than two billion people are infected with geohelminths, including the *Ascaris* (common roundworm), *Necator*, *Ancylostoma* (hookworms) and *Trichuris* (whipworm), mainly in underprivileged areas of the world [1]. *Trichuris trichiura* is a very common parasite of humans in developing countries, and causes trichuriasis in ∼ 600 million people worldwide, mainly in children aged between 5 and 15 years [2]. Trichuriasis can be associated with intestinal symptoms, such as abdominal pain, dysentery, nausea, vomiting, anorexia, constipation and chronic appendiceal syndrome [2]. Whipworms also infect a broad range of other hosts, including pigs (*T. suis*), dogs (*T. vulpis*), sheep (*T. ovis*), goats (*T. skrjabini*), rats (*T. muris*) and non-human primates, and can cause clinical disease similar to trichuriasis of humans [3]–[7].

*Trichuris* infects non-human primates in many countries, including Belgium [7], China [8], Ethiopia [9], Kenya [10], [11], Peru [12], South Africa [13]. In spite of the high prevalence of *Trichuris* sometimes reported in non-human primates [13], it is not clear whether the non-human primates harbour *T. trichiura* or other congeners. Based on morphological features of adult worms, *Trichuris* of non-human primates (including *Trichuris cynocephalus* and *T. rhinopithecus*) have been regarded as *T. trichiura* [14], [15]. However, the identification of *Trichuris* to species using morphological criteria alone is not reliable. Moreover, neither larval or egg stages of *Trichuris* from humans, pigs and non-human primates can be identified or differentiated unequivocally to species using classical diagnostic approaches [14], [16]. Therefore, there is a need for suitable molecular approaches to accurately identify and distinguish closely-related *Trichuris* species from different hosts.

Molecular tools, using genetic markers in mitochondrial (mt) DNA and in the internal transcribed spacer (ITS) regions of nuclear ribosomal DNA (rDNA), have been used effectively to identify nematode species [17]–[21]. For whipworms, mtDNA has been used in China to show clear genetic distinctiveness between human- and pig-derived *Trichuris* [22], and between *T. ovis* and *T. discolor* from ruminant hosts [23]. Using ITS rDNA, recent studies of *Trichuris* specimens obtained from humans and pigs [24], [25] also indicate that *T. trichiura* and *T. suis* are separate species. Cutillas et al. [26] used the ITS rDNA to infer the existence of two separate *Trichuris* species in murid and arvicolid rodents. In other studies from Spain, ITS rDNA has also been employed to distinguish among *T. suis* from swine, *T. vulpis* from dogs [27] and *T. trichiura* from the non-human primates (i.e. *Pan troglodytes*, *Colobus guereza kikuyensis* and *Nomascus gabriellae*) [15]. Although a recent investigation has shown two distinct *Trichuris* genotypes infecting both humans and non-human primates [28], there is still a paucity of information on *Trichuris* from different species of primates and countries around the world. Therefore, in the present study, we characterized the mt genomic and ITS rDNA sequences of *Trichuris* from the endangered François’ leaf-monkey (*Trachypithecus (Presbytis) françoisi*), which usually lives in close proximity to human populations in southern China [29], and we compared them with homologous sequences of human- and pig-derived *Trichuris*, and then tested the hypothesis that this monkey-derived *Trichuris* is a separate species.

## Materials and Methods

### Ethics Statement

This study did not require approval by an ethics committee. Two François’ leaf-monkeys, from which *Trichuris* specimens were collected from their caeca *post-mortem*, were handled and housed in a zoo in strict accordance with good animal practices required by the Animal Ethics Procedures and Guidelines of the People’s Republic of China. The monkeys were caged, and there were two rooms in a cage; one was indoor and the other was outdoor. They were fed fruits and vegetables. The monkeys were under the care and treatment of a licensed veterinarian at the zoo, and were euthanized due to acute gastric dilation.

### Parasites and Isolation of Total Genomic DNA

Two adult specimens of *Trichuris* (designated “monkey-*Trichuris*”) were collected from each of the two François’ leaf-monkeys, and were washed in physiological saline, identified morphologically [14], fixed in 70% (v/v) ethanol and stored at –20°C until use. Total genomic DNA was isolated separately from four individual worms (coded TH1-TH4) using an established method [30].

### Long-range PCR-based Sequencing of mt DNA

To obtain some mt sequence data for primer design, we PCR-amplified regions (400–500 bp) of the *cox*1 gene by using a (relatively) conserved primer pair JB3-JB4.5 [31], and *nad*5 gene was amplified using primers NAD5F (forward; 5′- CAAGGATTTTTTTGAGATCTTTTTC-3′) and NAD5R (reverse; 5′- TAAACCGAATTGGAGATTTTTGTTT-3′) designed to conserved regions of sequence between human- and pig-derived *Trichuris*. The amplicons were sequenced in both directions using BigDye terminator v.3.1, ABI PRISM 3730. We then designed primers (Table 1) to regions within *cox*1, *nad*5 and *rrn*L (based on sequences conserved between human- and pig-derived *Trichuris*) and amplified from total genomic DNA (from an individual worm, coded TH1) the entire mt genome in three overlapping fragments (each ∼ 5 kb): *cox*1*-nad*5, *nad*5-*rrn*L and *rrn*L-*cox*1. The cycling conditions used were 92°C for 2 min (initial denaturation), then 92°C/10 s (denaturation), 48–52°C/30 s (annealing), and 60°C/10 min (extension) for 10 cycles, followed by 92°C/10 s, 58–52°C/30 s, and 60°C/10 min for 20 cycles, with a cycle elongation of 10 s for each cycle and a final extension at 60°C/10 min. Each amplicon, which represented a single band in a 0.8% (w/v) agarose gel, following electrophoresis and ethidium-bromide staining, was column-purified and then sequenced using a primer walking strategy [32].

**Table 1:** Sequences of primers used to amplify mitochondrial DNA regions from monkey-*Trichuris*.

Primer	Sequence (5′ to 3′)
THCO1F	GTTTTCTCTCTGGACCGATTACCTA
THND5R	TACTTTAGTAGTTGCAGGGGTTATC
THND5F	TAGGAGCAGCCATAGCGATAGGTAA
TH16SR	AATCACGTAATGTTCCATCGTCGAA
TH16SF	TAAACGAGAAGACCCTAGGAACTTG
THCO1R	CGAAAAGTATGTATATGGTGCCAAT

### Sequence Analyses

Sequences were assembled manually and aligned against the complete mt genome sequences of *T. trichiura* [22] using the computer program Clustal X 1.83 [33] to infer gene boundaries. The open reading frames (ORFs) were identified using ORFFinder (http://www.ncbi.nlm.nih.gov/gorf/gorf.html) employing the invertebrate mitochondrial code, and subsequently compared with that of *T. trichiura* [22]. Translation initiation and termination codons were identified based on comparison with those reported previously [22]. The secondary structures of 22 tRNA genes were predicted using tRNAscan-SE [34] and/or manual adjustment [35], and rRNA genes were identified by comparison with those known for *Trichuris* [22].

### Sequencing of ITS rDNA and mt *rrn*L

The full ITS rDNA region including primer flanking 18S and 28S rDNA sequences was PCR-amplified from individual DNA samples using universal primers NC5 (forward; 5′-GTAGGTGAACCTGCGGAAGGATCATT-3′) and NC2 (reverse; 5′-TTAGTTTCTTTTCCTCCGCT-3′) described previously [36]. The primers *rrn*LF (5′-AAAACTCGGCAAATCGCATACTAAT-3′) and *rrn*LR (5′- CGAGCCACAAGACAGTAATGATAAG -3′) designed to conserved mt genome sequences within the *rrn*L gene were employed for PCR amplification and subsequent sequencing of a portion (∼ 600 bp) of this gene from multiple individuals of monkey-derived *Trichuris*.

### Phylogenetic Analyses

Amino acid sequences inferred from the 12 protein-coding genes (i.e. not *atp*-8) common among all of the nematodes included here were concatenated into a single alignment, and then aligned with those of four other enoplid nematodes (GenBank accession nos. GU385218, GU070737, JQ996232 and JQ996231 for *T. trichiura*, *T. suis*, *T. ovis* and *T. discolor*, respectively), using *T. spiralis* (accession no. NC_002681) [37] as an outgroup. Ambiguous sites and regions in the alignment were excluded using Gblocks (http://molevol.cmima.csic.es/castresana/Gblocks_server.html) [38] using default parameters. The *rrn*L sequences determined here and those of human- and pig-derived *Trichuris* [22] were aligned and subjected to phylogenetic analysis using *Trichinella spiralis* (accession no. NC_002681) [37] as an outgroup (cf. [39]). Phylogenetic analyses were conducted using Bayesian inference (BI), as described previously [40]. Phylograms were drawn using the program Tree View v.1.65 [41].

## Results

### Features of the Circular mt Genome of *Trichuris* from the François Leaf-monkey

**Figure 1:**
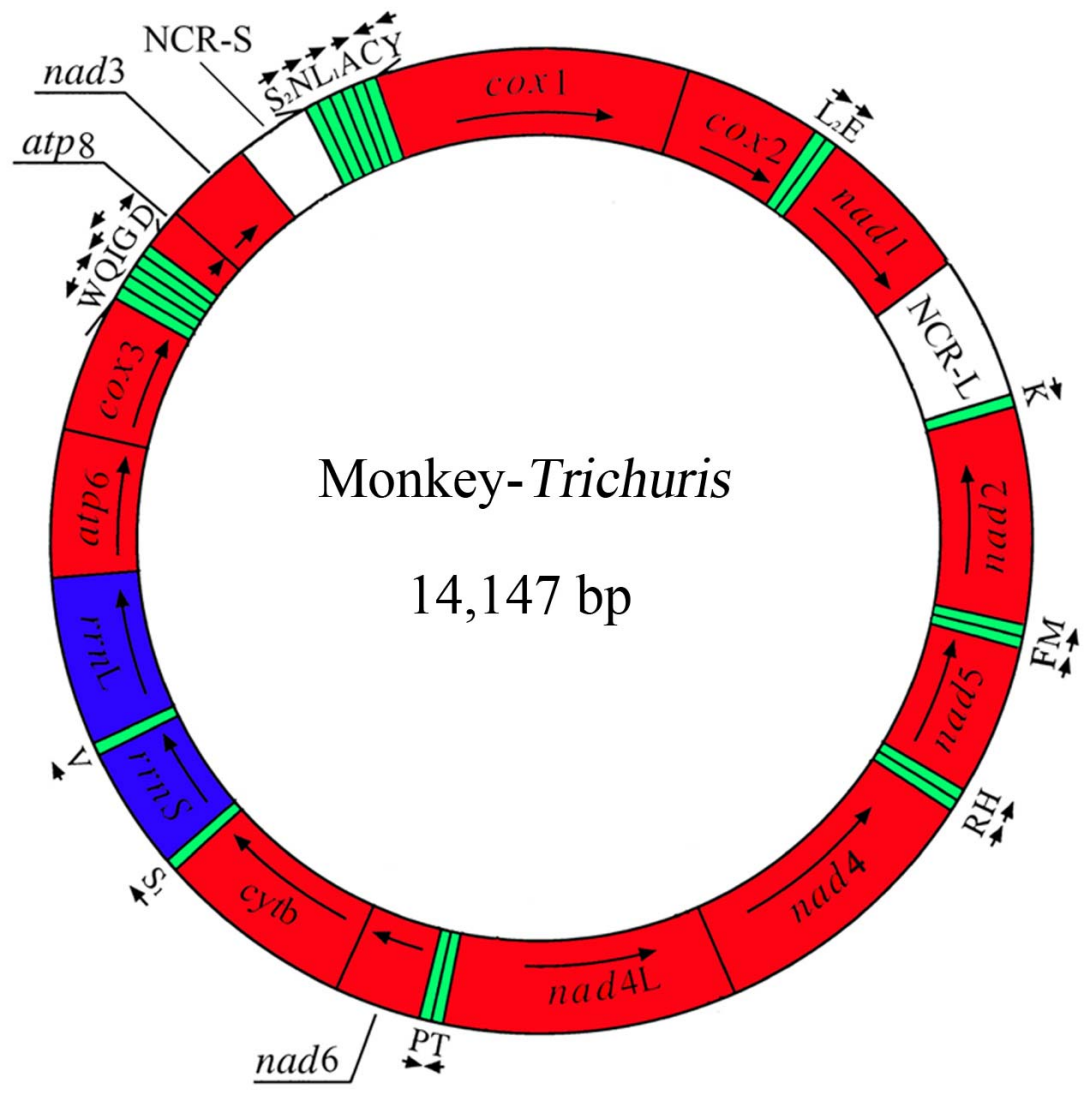
Structure of the mitochondrial genome for *Trichuris* from the François’ langur (*Trichuris* sp.). Genes are designated according to standard nomenclature, except for the 22 tRNA genes, which are designated using one-letter amino acid codes, with numerals differentiating each of the two leucine- and serine-specifying tRNAs (L1 and L2 for codon families CUN and UUR, respectively; S1 and S2 for codon families AGN and UCN, respectively). “NCR-L” refers to a large non-coding region; “NCR-S” refers to a small non-coding region.

The complete mt genome sequence was 14,147 bp in length (GenBank accession no. KC461179). The mt genome contains 13 protein-coding genes (*cox*1-3, *nad*1-6, *nad*4L, *cyt*b, *atp*6 and *atp*8), 22 transfer RNA genes and two ribosomal RNA genes (*rrn*S and *rrn*L) (Table 2); the *atp*8 gene is encoded (Figure 1). The protein-coding genes are transcribed in different directions, as reported for *T. trichiura* and *T. suis* [22] (Table 2). Protein-coding genes were annotated by aligning sequences, and identifying translation initiation and termination codons by comparison with homologous sequences for other whipworms (Table 2).

**Table 2:** Positions and nucleotide sequence lengths of mitochondrial genome of monkey*-Trichuris* (MT) compared with that of human*-Trichuris (T. trichiura = *TT*)* and pig*-Trichuris* (*T. suis = *TS).

Gene or region	Positions and nt sequence lengths (bp)			Strand	Initiation/termination codons			Anticodons
	MT	TT	TS		MT	TT	TS	MT/TT/TS
*cox*1	1–1536	1–1545	1–1542	H	ATG/TAG	ATG/TAA	ATG/TAG	
*cox*2	1556 –2230	1560 –2234	1578 –2258	H	ATG/TAA	ATG/TAA	ATG/TAA	
tRNA–LeuUUR (L_2_)	2264–2328 (65)	2251–2313 (63)	2271–2332 (62)	H				TAA
tRNA–Glu (E)	2339–2396 (58)	2318–2374 (57)	2337–2393 (57)	H				TTC
*nad*1	2413–3312	2397–3296	2415–3314	H	ATT/TAA	ATA/TAA	ATT/TAG	
Non–coding region (NCR–L)	3313–3436 (124)	3297–3458 (162)	3315–3458 (144)	H				
tRNA–Lys (K)	3437–3501 (65)	3459–3524 (66)	3459–3521 (63)	H				TTT
*nad*2	3508–4407	4406–3522	4414–3533	L	ATT/TAG	ATA/TAA	ATA/TAG	
tRNA–Met (M)	4468–4408 (61)	4479–4419 (61)	4485–4424 (62)	L				CAT
tRNA–Phe (F)	4463–4521 (59)	4530–4474 (57)	4546–4488 (59)	L				GAA
*nad*5	6070–4523	6078–4531	6094–4538	L	ATA/TAA	ATA/TAA	ATA/TAG	
tRNA–His (H)	6061–6122 (62)	6128–6072 (57)	6150–6094 (57)	L				GTG
tRNA–Arg (R)	6188–6124 (65)	6194–6130 (65)	6218–6152 (67)	L				ACG
*nad*4	7401–6190	7406–6195	7432–6224	L	ATG/TAA	ATG/TAA	ATA/TAA	
*nad*4L	7420–7680	7682–7425	7901–7650	L	ATA/TAA	ATA/TAA	ATA/TAG	
tRNA–Thr (T)	7685–7744 (60)	7687–7744 (58)	7905–7962 (58)	H				TGT
tRNA–Pro (P)	7796–7743 (54)	7802–7744 (59)	8023–7966 (58)	L				TGG
*nad*6	7798–8265	7795–8271	8016–8486	H	ATG/TAA	ATT/TAA	ATT/TAA	
*cyt*b	8273–9379	8278–9384	8501–9613	H	ATG/TAG	ATG/TAG	ATG/TAG	
tRNA–Ser AGN (S1)	9378–9429 (52)	9383–9432 (50)	9612–9666 (55)	H				GCT
*rrn*S	9422–10126	9425–10122	9664–10375	H				
tRNA–Val (V)	10129–10185 (57)	10124–10180 (57)	10375–10431 (57)	H				TAC
*rrn*L	10192–11198	10180–11190	10440–11450	H				
*atp*6	11175–11996	11173–12000	11422–12249	H	ATA/TAA	ATA/TAA	ATA/TAA	
*cox*3	12001–12744	11975–12748	12259–13035	H	ATG/TAA	ATG/TAA	ATG/TAA	
tRNA–Trp (W)	12844–12779 (66)	12817–12755 (63)	13106–13040 (67)	L				TCA
tRNA–Gln (Q)	12852–12908 (57)	12821–12874 (54)	13110–13166 (57)	H				TTG
tRNA–Ile (I)	12992–12927 (66)	12937–12871 (66)	13234–13169 (66)	L				GAT
tRNA–Gly (G)	13057–12996 (62)	13003–12947 (57)	13308–13253 (56)	L				TCC
tRNA–Asp (D)	13063–13121 (59)	13009–13067 (58)	13302–13363 (62)	H				GTC
*atp*8	13109–13273	13055–13219	13360–13530	H	ATA/TAG	ATA/TAG	TTG/TAA	
*nad*3	13286–13624	13229–13570	13555–13896	H	ATT/TAA	ATA/TAA	ATA/TAA	
Non–coding region (NCR–S)	13625–13729 (105)	13571–13663 (93)	13887–14003 (117)	H				
tRNA–Ser UCN (S2)	13730– 13782 (53)	13664–13715 (52)	14004–14055 (52)	H				TGA
tRNA–Asn (N)	13782–13836 (55)	13715–13768 (54)	14055–14113 (59)	H				GTT
tRNA–Leu CUN (L1)	13841–13907 (67)	13776–13842 (67)	14131–14193 (63)	H				TAG
tRNA–Ala (A)	13924–13983 (60)	13845–13899 (57)	14196–14250 (55)	H				TGC
tRN A–Cys (C)	14065–14003 (63)	13979–13925 (55)	14274–14328 (55)	L				GCA
tRNA–Tyr (Y)	14127–14068 (60)	14046–13986 (50)	14394–14336 (59)	L				TGT

Twenty-two tRNA genes, which varied from 52 to 67 bp in length, were predicted from the mt genomes. The two ribosomal RNA genes (*rrn*L and *rrn*S) were inferred; *rrn*L is located between tRNA-Val and *atp*6, and *rrn*S is located between tRNA-Ser ^(AGN)^ and tRNA-Val. The lengths of *rrn*L and *rrn*S are 1,007 bp and 705 bp, respectively. The A+T contents of *rrn*L and *rrn*S are 69.02% and 70.21%, respectively.

Two AT-rich non-coding regions (NCRs) were inferred in the mt genome. For this genome, the long NCR (designated NCR-L; 124 bp in length) is located between the *nad*1 and tRNA-Lys (Figure 1), has an A+T content of 59.68%. The short NCR (NCR-S; 105 bp in length) is located between genes *nad*3 and tRNA-Ser ^(UCN)^ (Figure 1), with an A+T content of 79.25%.

### Nuclear Ribosomal DNA Regions of *Trichuris* from the Monkey

The rDNA region including ITS-1, ITS-2 and intervening 5.8 rRNA gene sequenced from individual *Trichuris* samples (coded TH1-TH4) was 1,314 bp in length. Individual spacers were 570 bp (ITS-1) and 468 bp (ITS-2), and the 5.8S rRNA gene was 154 bp long.

### Comparative Analyses Among Monkey-*Trichuris*, Human-*Trichuris* and Pig-*Trichuris*

The mt genome sequence of monkey-*Trichuris* (accession no. KC461179) was 14,147 bp in length, 101 bp longer than that of human-*Trichuris*, and 289 bp shorter than that of pig-*Trichuris*. The arrangement of the mt genes (i.e., 13 protein genes, 2 *rrn* genes and 22 tRNA genes) and NCRs were the same. A pairwise comparison of the nucleotide sequences of each mt gene and the amino acid sequences conceptually translated from individual protein genes was made among the three taxa of *Trichuris* (from the three host species) (Table 3). The sequence lengths of individual genes varied among these taxa, except for the *nad*1 gene, which was the same (Table 3). The magnitude of sequence variation in each gene among the three taxa of *Trichuris* ranged from 24.2–50.9% for nucleotide sequences and 13.6–62.5% for amino acid sequences (Table 3). The sequence difference across the entire mt genome between monkey- and human-*Trichuris* was 29.35% (a total of 4,152 nucleotide alterations). This difference across the entire mt genome between monkey- and pig-*Trichuris* was 33.49% (a total of 4835 nucleotide alterations). The greatest variation among the three taxa of *Trichuris* was in the *atp*8 gene (42.4–58.9%), whereas least differences (24.3%–31.5%) were detected in the *rrn*S and *rrn*L subunits, respectively (Table 3).

**Table 3:** Nucleotide and/or predicted amino acid (aa) sequence differences for mt protein-coding and ribosomal RNA genes among monkey*-Trichuris* (MT), human*-Trichuris* (*Trichuris trichiura = *TT) and pig*-Trichuris* (*T. suis* = TS).

Gene/region	Nucleotide length (bp)			Nucleotide difference (%)			Number of aa			aa difference (%)		
	MT	TT	TS	MT/TT	MT/TS	TT/TS	MT	TT	TS	MT/TT	MT/TS	TT/TS
*atp*6	822	828	828	36.8	40.2	39.6	273	275	275	40.0	49.5	49.5
*nad*1	900	900	900	29.6	32.8	33.1	299	299	299	28.1	32.4	33.1
*nad*2	900	885	882	34.7	39.2	34.1	299	294	293	31.8	44.2	40.8
*nad*3	339	342	342	32.7	38.6	33.6	112	113	113	27.4	35.4	31.9
*nad*4	1212	1212	1209	31.3	41.2	40.9	403	403	402	31.3	55.1	58.3
*nad*4L	261	258	252	28.7	35.6	38.0	86	85	83	36.0	46.5	42.4
*nad*5	1548	1548	1557	34.6	38.6	35.9	515	515	518	37.5	47.1	42.5
*nad*6	468	477	471	32.3	35.7	33.1	155	158	156	35.4	45.6	38.6
*cox*1	1536	1545	1542	24.2	25.0	25.4	511	514	513	13.6	16.6	13.6
*cox*2	675	675	681	24.3	31.6	30.7	224	224	226	18.7	31.0	28.8
*cox*3	774	774	777	29.2	31.0	35.5	257	257	258	25.7	30.6	34.5
*cyt*b	1107	1107	1113	26.1	29.0	27.7	368	368	370	20.9	27.3	26.2
*atp*8	165	165	171	42.4	50.9	47.4	54	54	56	55.6	58.9	62.5
*rrn*S	705	698	712	25.4	24.3	24.6	–	–	–	–	–	–
*rrn*L	1007	1011	1011	25.1	31.5	25.1	–	–	–	–	–	–

**Figure 2:**
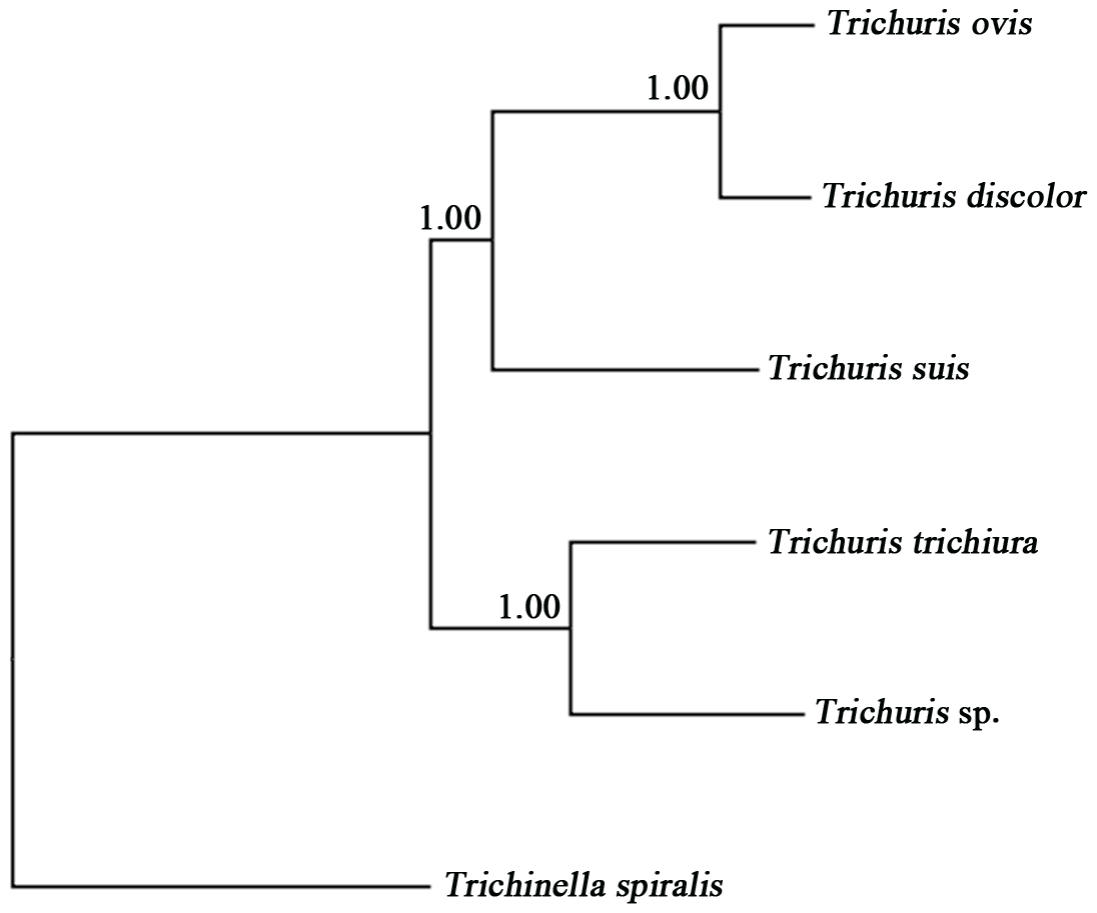
Inferred phylogenetic relationship of *Trichuris* from the François’ langur (*Trichuris* sp.) with *T. trichiura* , *T. suis*, *T. ovis* and *T. discolor*. Analysis of the concatenated amino acid sequence data representing 12 protein-coding genes (with the exception of *atp*8) by Bayesian inference (BI), using *Trichinella spiralis* (NC_002681) as the outgroup.

Amino acid sequences inferred from individual mt protein genes of monkey-*Trichuris* were compared with those of human- and pig-*Trichuris*. The difference across amino acid sequences of the 13 protein genes between the monkey- and human-*Trichuris* was 28.52% (a total of 1015 amino acid alterations) and 38.28% (a total of 1364 amino acid alterations) between the monkey- and pig-*Trichuris*, respectively. The amino acid sequence differences among three taxa of *Trichuris* ranged from 13.6–62.5%, with COX1 being the most conserved and ATP8 the least conserved protein. Phylogenetic analyses of concatenated amino acid sequence data sets, using *T. spiralis* as an outgroup, revealed that the monkey-*Trichuris* was more closely related to the human-*Trichuris* than to representative *Trichuris* species from porcine and ruminant hosts, with absolute support (pp = 1.00) (Figure 2).

**Figure 3:**
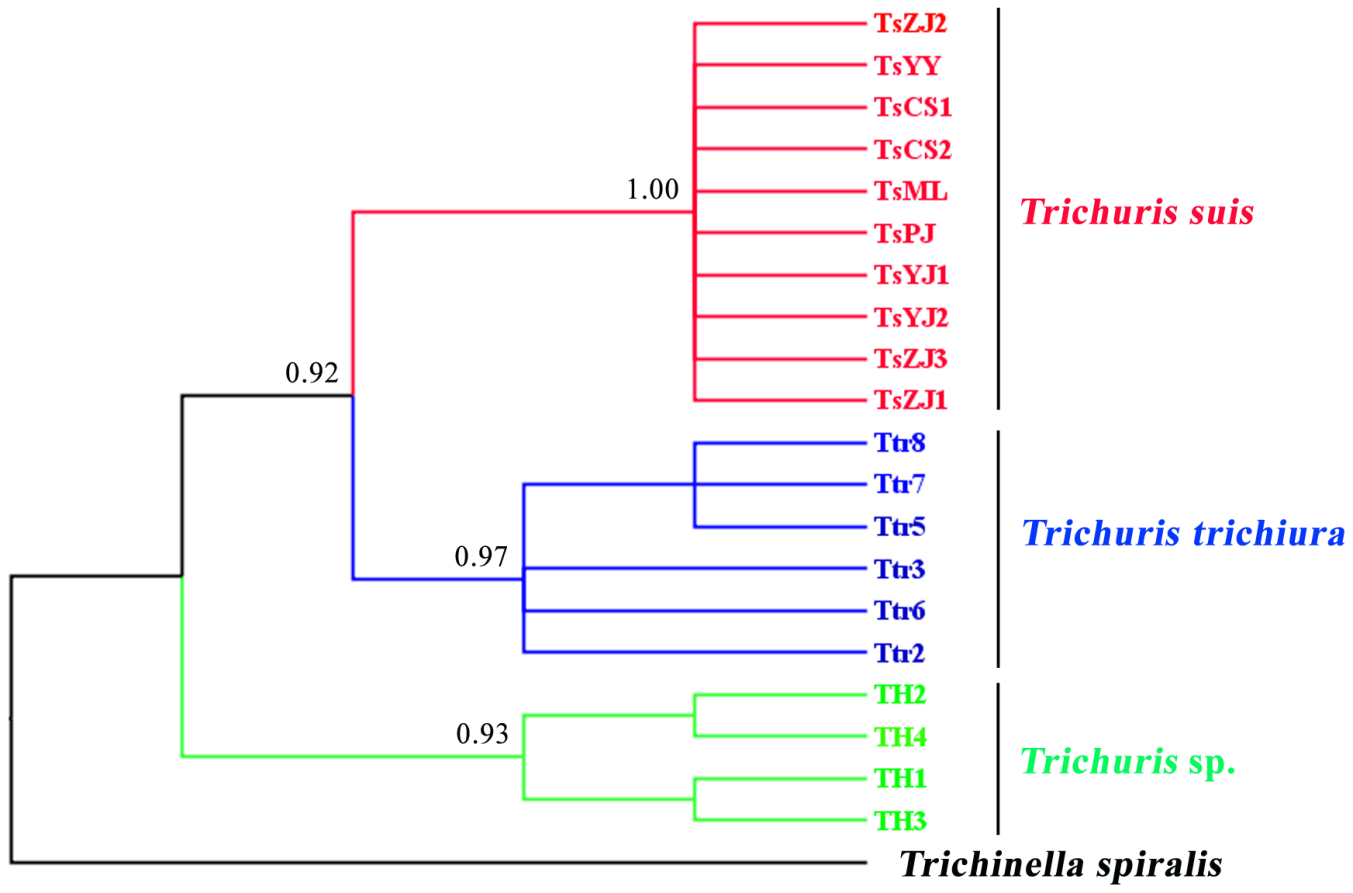
Inferred genetic relationships of four individual specimens of from the François’ langur (*Trichuris* sp.) with those of *Trichuris trichiura* (n = 6) and *T. suis* (n = 10) from China. The analyses of mitochondrial *rrn*L sequence data were carried out by Bayesian inference (BI), using *Trichinella spiralis* as the outgroup. Posterior probabilities (pp) values of <0.9 are not shown.

Comparison of the mt genomes of monkey-*Trichuris*, human-*Trichuris* and pig-*Trichuris* showed that the *rrn*S and *rrn*L were the two most conserved genes (Table 3). Sequence variation in part of the *rrn*L gene was assessed among four individuals of *Trichuris* from monkeys. The *rrn*L sequences of the four monkey-*Trichuris* individuals (GenBank accession nos. KC481232-KC481235) were of the same length (616 bp). Nucleotide variation among the four monkey-*Trichuris* individuals was detected at 15 sites (15/616; 2.44%). The four monkey-*Trichuris* sequeces were aligned with 10 and six *rrn*L sequences (GenBank accession nos. AM993017-AM993032; [22]) reported previously for human- and pig- derived *Trichuris*, respectively. The alignment of the partial *rrn*L sequences revealed that all individuals of monkey-*Trichuris* differed at 140 nucleotide positions (140/430; 32.6%) when compared with human- and pig-*Trichuris*. Phylogenetic analysis of the *rrn*L sequence data from individual worms revealed strong support for the separation of monkey-*Trichuris* from human-*Trichuris* and pig-*Trichuris* (Figure 3).

Sequence variation was examined for both ITS-1 and ITS-2 of monkey-*Trichuris*. The ITS-1 and ITS-2 sequences from four individual adults of monkey-*Trichuris* were compared with those of human- and pig-derived *Trichuris* [25]. Sequence variations were 0–0.7% (ITS-1) and 0–0.9% (ITS-2) among the three specimens of monkey-*Trichuris*. However, the sequence differences were 21.4–22.3% (ITS-1) and 22.4–23.7% (ITS-2) between the monkey- and human-*Trichuris*, and 56.5–57.0% (ITS-1) and 43.6–45.5% (ITS-2) between the monkey- and pig-*Trichuris*.

## Discussion

To date, more than 20 *Trichuris* species have been described from various mammalian hosts based on the microscopic features of the adult worms [42]. Some studies (e.g., [43], [44]) have claimed that male spicule and body lengths are useful morphological parameters for the differentiation of *Trichuris* species. However, other studies have shown that these measurements are not necessarily reliable for specific identification [15]. For instance, Cutillas et al. (2009) [15] observed that the spicule lengths of *T. trichiura* and *T. suis* overlapped. While other workers considered that the presence of pericloacal papillae might be useful for species determination [45], also this criterion does not appear to allow accurate identification/delineation [15]. Clearly, these studies show that morphological characters or morphometrics should be interpreted with caution. For this reason, we employed here a molecular genetic approach, logically extending previous studies [22]–[26], so that comparative genetic analyses could be conducted.

The present investigation shows clear genetic distinctiveness between *Trichuris* from the François’ langur and *Trichuris* from humans and livestock animals (i.e., *T. suis*, *T. ovis* and *T. discolor*) (Figure 2). Our and previous findings [22]–[27] support the contention that each *Trichuris* species has a very specific affiliation with a particular host species [16], although, to date, only small numbers of adult worms have been studied molecularly. Clearly, larger population genetic and molecular epidemiological studies should be conducted using the mt and nuclear markers defined in this and previous studies [22]–[28] to further test this hypothesis.

The sequence difference in the inferred mt proteome between monkey- and human-*Trichuris* was 29.4%, and sequence variation among individual worms from each host species was low (0–2.4%), suggesting that these parasites are separate species. This proposal was further supported by phylogenetic analysis (cf. Figure 3). Previous studies [7]–[13] have indicated that many non-human primates, such as *Colobus guereza*, *Macaca fascicularis*, *M. silenus*, *Papio anubis*, *P. hamadryas ursinus* and *Theropithecus gelada*, can harbour *Trichuris*. However, the specific identity, host specificity and zoonotic potential of each operational taxonomic unit (OTU) [13] of *Trichuris* from each of these host species are unknown. Based on molecular findings to date for *Trichuris* from other animal species [22]–[28], [46]–[51], we anticipate that each primate species harbours its own species of *Trichuris*, but, clearly, this proposal requires rigorous testing.

*Trachypithecus françoisi* is a threatened/endangered species of primate nearing extinction [52]. Populations of this langur have been on the decline for the past 30 years. For instance, the populations in Guangxi province, China, crashed from 4000–5000 individuals in 1980 to a mere 307 in 2002–2003 [53]. The main factors linked to this decline have been hunting, habitat destruction and harvesting of langur organs for the preparation of traditional medicines [54], [55]. Another likely threat to the François’ langur, particularly in captive situations in conservation parks and zoos, is whipworm disease. This statement is supported by reports from China (e.g., [56]–[59]), indicating that *Trichuris* infection is common (14.3–100%) in this langur in zoos and conservation parks, as are clinical cases of trichuriasis. The direct life cycle of *Trichuris*, the accumulation of eggs in environments with relatively high population density of primates (animals) and the robustness and longevity of the infective stage (larvated eggs) in the environment [60] are all factors that contribute significantly to a gradual increase of trichuriasis in ‘closed’ environments, such as parks [61]. Although we expect the monkey-*Trichuris* studied herein to be specific to the François’ langur, there is a possibility that this parasite is transmissible to other primates, including humans. However, this proposal needs to be assessed.

In spite of molecular evidence for the existence of a unique *Trichuris* species in the endangered François’ langur, the interpretations from the present study are guarded, at this stage, until detailed population genetic investigations have been conducted. Future studies should include (i) exploring, in detail, nucleotide variation in rDNA and mtDNA within and among *Trichuris* populations from a range of different primate species and countries, and to establish whether more than one *Trichuris* species infect non-human primates, (ii) establishing, using accurate molecular tools, whether cross-host species infection occurs or not, (iii) undertaking detailed morphological studies, by scanning electron microscopy and field emission scanning electron microscopy, of whipworms from various non-humans primates. This focus is important because, traditionally, the diagnosis of *Trichuris* infection in animals has relied mainly on the morphological identification of adult and egg stages.
